# Timing of operation for poor‐grade aneurysmal subarachnoid hemorrhage: Relationship with delayed cerebral ischemia and poor prognosis

**DOI:** 10.1111/cns.14088

**Published:** 2023-01-10

**Authors:** Junlin Lu, Liang Wang, Runting Li, Fa Lin, Yu Chen, Debin Yan, Jun Yang, Ruinan Li, Zhipeng Li, Haibin Zhang, Heze Han, Kexin Yuan, Ke Wang, Yihang Ren, Xiaolin Chen, Yuanli Zhao, Jizong Zhao

**Affiliations:** ^1^ Department of Neurosurgery, West China Hospital Sichuan University Chengdu China; ^2^ Department of Neurosurgery Tianjin fifth Central Hospital Tianjin China; ^3^ Department of Neurosurgery, Beijing Tiantan Hospital Capital Medical University Beijing China; ^4^ China National Clinical Research Center for Neurological Diseases Beijing China; ^5^ Stroke Center Beijing Institute for Brain Disorders Beijing China; ^6^ Beijing Key Laboratory of Translational Medicine for Cerebrovascular Disease Beijing China; ^7^ Beijing Translational Engineering Enter for 3D Printer in Clinical Neuroscience Beijing China

**Keywords:** delayed cerebral ischemia, prognosis, subarachnoid hemorrhage, timing of surgery

## Abstract

**Aims:**

To assess differences in the clinical prognosis between different treatment timings in poor‐grade (Hunt and Hess grade 4–5) aneurysmal subarachnoid hemorrhage patients.

**Methods:**

The treated 127 poor‐grade aneurysmal subarachnoid hemorrhage patients were divided into three groups: early treatment within 2 days, treatment on days 3 to 10, and treatment for more than 10 days after the hemorrhage. Odd ratios with a 95% confidence interval were calculated in logistic regression for different timing strategies regarding delayed cerebral ischemia and poor prognosis at 3 months. Subgroup analyses were conducted to determine whether the different timing strategies affect the prognosis.

**Results:**

Patients who received the treatment on days 3 to 10 were prone to develop delayed cerebral ischemia and poor prognosis at 3 months. Postponing treatment in patients older than 55 years or diagnosed with an intraventricular hematoma on the initial computed tomography scan may lead to poor prognosis, with the early intervention group as a reference.

**Conclusions:**

Early intervention in poor‐grade aneurysmal subarachnoid hemorrhage is suggested to be implemented. The treatment on 3 to 10 days harbored the highest risk of poor prognosis; patients might benefit more from early intervention, especially for ones older than 55 years or diagnosed with an intraventricular hematoma.

## Graphical abstract

The ideal timing of coiling or clipping for poor‐grade aneurysmal subarachnoid hemorrhage is unknown. Within the Long‐term Prognosis of Emergency Aneurysmal Subarachnoid Hemorrhage trial, we assessed differences in the incidence of delayed cerebral ischemia and clinical prognosis between different timings of treatment. A total of 127 poor‐grade subarachnoid hemorrhage patients were divided into three categories: treatment ≤2 days, on days 3 to 10, and > 10 days after the hemorrhage. Logistic regression analysis for delayed cerebral ischemia and poor prognosis at 3 months for the different timing categories, with treatment ≤2 days as reference. Analyses were performed for all patients and were adjusted for baseline characteristics. Adjusted OR of delayed cerebral ischemia for treatment on days 3 to 10 was 2.44 (95% CI, 1.06–5.64) for all patients. Adjusted OR for poor prognosis at 3 months was 3.06 (95% CI, 1.27–7.39) for treatment at 3 to 10 days. Postponing treatment in poor‐grade aneurysmal subarachnoid hemorrhage patients may lead to delayed cerebral ischemia and poor prognosis. Our results support the current practice for early aneurysm treatment in poor‐grade subarachnoid hemorrhage patients. The treatment at 3 to 10 days harbored the highest risk of poor prognosis; patients might benefit more from early intervention for poor prognosis.

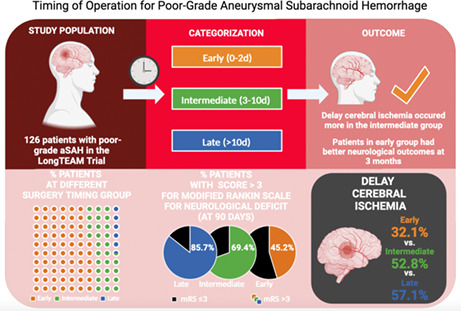

## 1 INTRODUCTION

Aneurysmal subarachnoid hemorrhage (aSAH) is a cerebrovascular disease characterized by remarkable morbidity and mortality.^1^, ^2^ Rebleeding after aSAH dramatically increases the risk of poor prognosis.^3^ Thus, treatment of the ruptured aneurysm is essential to prevent rebleeding and to improve the prognosis. Microsurgery in the initial 2 weeks was considerably risky because of the high rates of perioperative complications due to operating on the swollen and vulnerable brain tissue.^4^ Results of several studies over the 2000s suggested that patients undergoing early microsurgery (days 0–3) tended to have the best prognosis, while whether intermediate microsurgery (days 4–7) or late microsurgery has the worst prognosis remains uncertain.^5^, ^6^, ^7^

The past two decades have witnessed a burgeoning enthusiasm for endovascular treatment and a gradual shift in the modality of aneurysm treatment from traditional neurosurgical clipping to endovascular aneurysm occlusion.^8^, ^9^ A randomized study including 2106 patients treated by coiling or clipping demonstrated that early aneurysm treatment in subarachnoid hemorrhage patients is recommended; however, postponing treatment in patients eligible for treatment between days 5 and 10 after subarachnoid hemorrhage is not recommended.^4^ In fact, patients with poor‐grade aSAH (World Federation of Neurological Surgeons (WFNS) grading scale 4–5 or Hunt & Hess (H‐H) grade 4–5) accounted for only 5% of the entire cohort in this trial. Therefore, previous conclusions on timing of treatment might not be applied to poor‐grade aSAH patients since their population in the cohort study is low. Guidelines suggested that treatment of the ruptured aneurysm should be performed as early as feasible for most patients to reduce the rate of rebleeding after aSAH.^10^ Nevertheless, to date, the Handbook of Neurosurgery still recommends that H‐H grades 1 and 2 were operated upon as soon as an aneurysm was diagnosed while H‐H grade ≥ 3 managed until the condition improved to H‐H grade 2 or 1.^11^ It is reported that patients with poor‐grade aSAH can benefit from the early intervention of the aneurysm;^12^, ^13^, ^14^ however, the bias from the subjective stratification of timing and restriction on treating center or treatment type can be risky and gives rise to heterogeneous data. Hence, it is essential to investigate the optimal timing of aneurysm treatment for poor‐grade aSAH patients—whether the treatment should be delivered at an early date or postponed until after the 10th day. Moreover, it is worthwhile studying if the treatment modality influences optimal timing.

Within the Long‐term Prognosis of Emergency Aneurysmal Subarachnoid Hemorrhage (LongTEAM) trial (NCT04785976), we evaluated differences in the occurrence of delayed cerebral ischemia (DCI) and clinical prognosis between different timings of treatment for poor‐grade aSAH patients.

## 2 METHODS

### 2.1 Patients

We analyzed the data from patients who participated in the LongTEAM trial. The methods of this trial have been described previously.^15^ In short, patients were included in the trial if they had a definite aSAH within the previous 30 days. Most patients were in good clinical condition at admission and had anterior circulation aneurysms. All patients were managed according to the American Heart Association/American Stroke Association guidelines.^10^ Briefly, after being reviewed by both experienced cerebrovascular surgeons and endovascular specialists, patients received a multidisciplinary decision of treatment modalities based on the characteristics of the patient and their aneurysm. Microsurgical clipping may preferably be considered in patients with large intraparenchymal hematomas and middle cerebral artery aneurysms. Endovascular coiling is much likely to be considered for the elderly and those with aneurysms of the basilar apex.

The local institutional review boards approved the study with a waiver of consent. Secondary use of the registry data and additional review of medical records for this study were approved by institutional review boards. All the analyses were performed following the Declaration of Helsinki and the local ethics policies. The present study followed Strengthening the Reporting of Observational Studies in Epidemiology guidelines.

### 2.2 Perioperative management

Patient management conformed to guidelines set forth by the American Heart Association.^10^ All patients had angiographically documented aSAH confirmed by computed tomography (CT) or lumbar puncture. Once the patients were diagnosed with aSAH, regular CT angiography for vasospasm and CT perfusion for hypoperfusion were also conducted. Patients were routinely transferred to the intensive care unit after surgery. Between the time of aSAH symptom onset and aneurysm obliteration, a systolic blood pressure below 160 mm Hg was controlled with a titratable agent to reduce the risk of rebleeding. Nimodipine was administered intravenously centrally (1–2 mg/h infusion) but reduced if any difficulty was encountered in maintaining adequate blood pressure. Regarding unconscious patients and those who deteriorated postoperatively due to possible cerebral ischemia, induction of hypertension (intravenous norepinephrine) was conducted to achieve a systolic pressure of up to 160 mm Hg and maintained until 48 h after clinical improvement. A non‐contrast CT was usually conducted 6 h after surgery to determine if the hematoma was enlarged or de nova hemorrhage occurred. In addition, CT scans were repeated postoperatively for patients with deep coma to assess intracranial status.

### 2.3 Variable measurements

**FIGURE 1:**
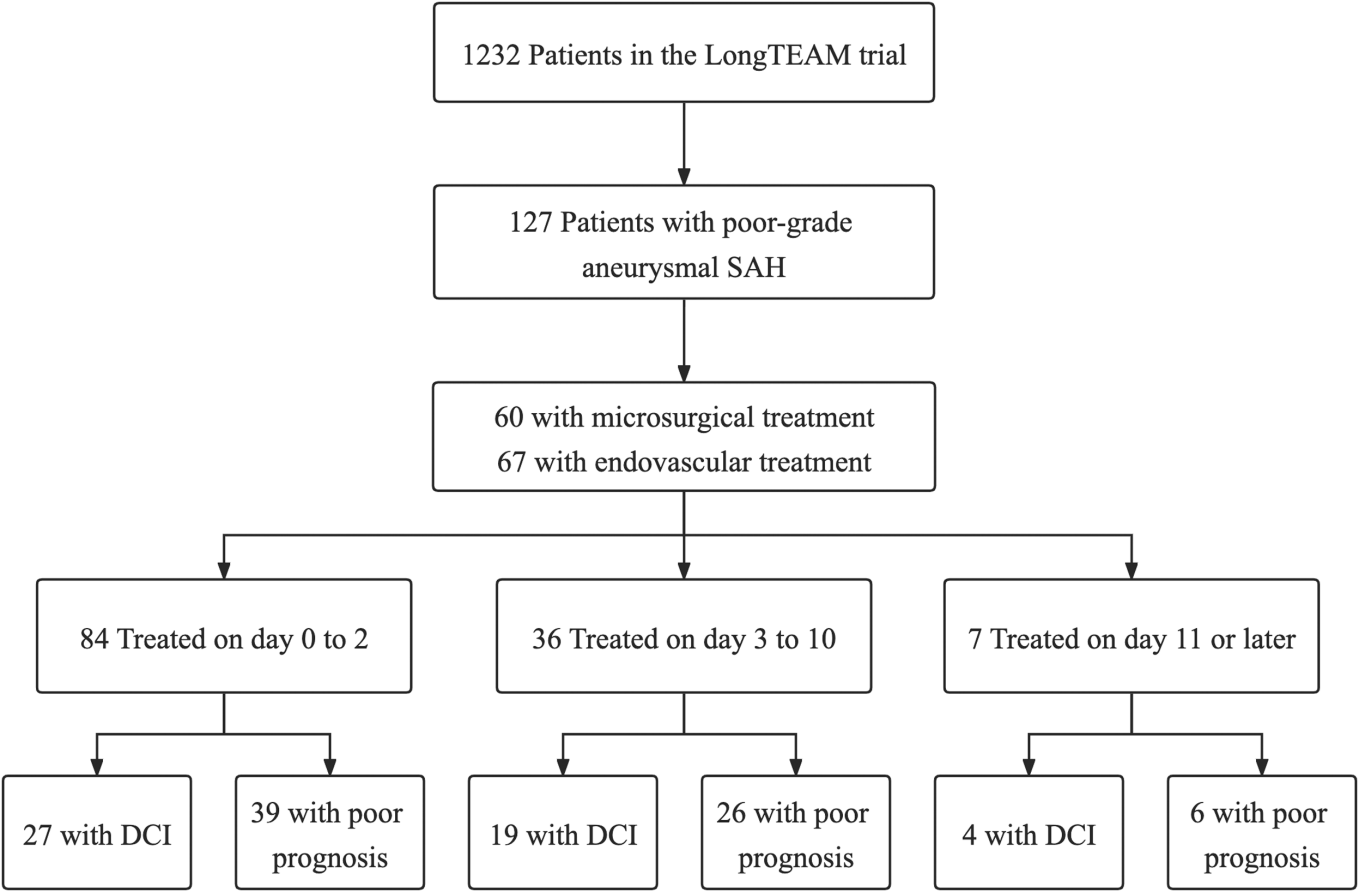
Flow diagram of the study population.

The following measurements were recorded for patients: age, sex, medical history, clinical grade on admission using the H‐H grade and WFNS grading scale, amount of blood on the initial CT according to the modified Fisher Scale (mFS),^16^ and the occurrence of DCI. For the current study, we analyzed patients with poor‐grade aSAH (H‐H grade 4–5) (Figure 1). Age was dichotomized at 55 years. According to the amount of cisternal blood and the presence of concomitant intraventricular hemorrhage on the first non‐contrast CT, the mFS was dichotomized into two groups, mFS 1–2 (thin focal or diffuse SAH, with or without intraventricular hemorrhage) and mFS 3–4 (thick SAH, with or without intraventricular hemorrhage). DCI was defined as clinical deterioration, the occurrence of a new focal neurologic deficit or a new infarction on CT not attributable to other causes.^17^, ^18^ The location of the aneurysm that leads to aSAH was assessed by the CT angiography or digital subtraction angiography on admission. The location of the aneurysm was dichotomized into anterior circulation aneurysms and posterior circulation aneurysms. Preoperative hydrocephalus and intraventricular hematoma were assessed regarding the CT scan on admission. Self‐reported questionnaires evaluated the clinical prognosis with the modified Rankin Scale (mRS) score at 3 months. Poor prognosis was defined as the mRS score higher than three or death.

### 2.4 Statistical analyses

**FIGURE 2:**
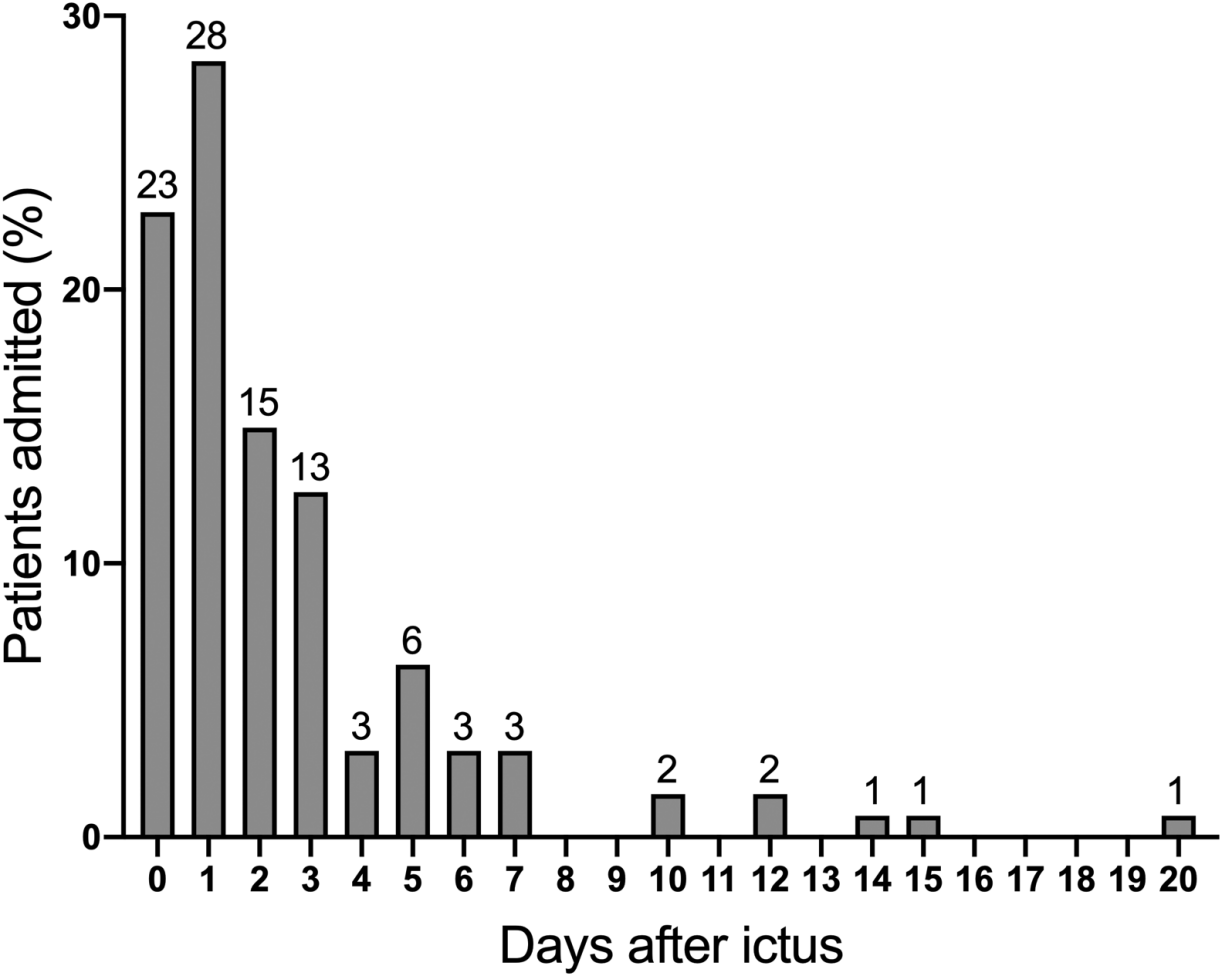
Distribution of days from onset to surgery for all patients diagnosed as having had an aneurysmal subarachnoid hemorrhage. (*n* = 22; day of aSAH = day 0).

The Kolmogorov–Smirnov and Shapiro–Wilk tests for normality were used to assess data distribution. Numeric variables are presented as percentages and continuous variables as median with interquartile range (skewed distribution). First, patients were divided into three groups according to the timing of treatment after the aSAH: within 2 days, on days 3 to 10, and >10 days. The distribution of days from onset to surgery for all patients diagnosed with an aSAH was presented in Figure 2. The logistic regression analysis was conducted to calculate the ORs with 95% CI for DCI between the different timing strategies. We performed these analyses separately for all patients, coiled and clipped patients. In addition, ORs with 95% CI were calculated in logistic regression for poor prognosis at 3 months between different timing strategies. To further determine the effect of treatment timing on prognosis within different groups, we conducted subgroup analyses by treatment modalities, age, preoperative hydrocephalus, intraventricular hematoma, and external ventricular or lumbar drainage treatment. All analyses were adjusted for other confounding factors, such as age, sex, clinical condition at admission, amount of blood on the initial CT scan, and the location of the aneurysm. The Kruskal–Wallis test and multiple comparisons were performed to analyze the difference between mRS scores at 3‐month follow‐up of patients for the different timing strategies. Statistical significance was set at *p* < 0.05 for 95% CI. Data were analyzed using IBM SPSS Statistics version 26.0 (IBM Corp.).

## 3 RESULTS

Of the 1232 patients enrolled in the LongTEAM trial, 127 (mean age 57 years; 73 female) were diagnosed with poor‐grade aSAH. Of these patients, 84 (84/127, 66.1%) patients were treated on days 0 to 2 after aSAH, 36 (36/127, 28.3%) patients were treated on days 3 to 10, and 7 (7/127, 5.6%) patients were treated on day 11 or later (Table 1). Most of these patients had a large amount of blood on the initial CT scan. Patients were more likely to receive the endovascular treatment within 10 days after aSAH. No significant difference in the overall proportion of patients was observed between the neurosurgical and endovascular treatment groups. In addition, patients in the later treatment windows did not perform a higher incidence of vasospasm and hypoperfusion than those in the earlier windows, according to the CT angiography and CT perfusion on admission. Rebleeding before aneurysm treatment was more frequently observed in the group treated after day 10.

**TABLE 1:** Baseline and disease characteristics of the 127 included patients.

	Timing of treatment, days after SAH			
Characteristic	All	0–2	3–10	>10
No. of pts	127	84	36	7
Age (years, median with IQR)	60 (51–65)	60 (51–66)	57 (52–64)	50 (45–63)
Female sex (%)	73 (57.5)	48 (57.1)	22 (61.1)	3 (42.9)
% w/History of hypertension	84 (66.1)	58 (69.0)	23 (63.9)	3 (42.9)
Current smoker (%)	42 (33.0)	29 (34.5)	12 (33.3)	1 (14.3)
WFNS score (%)				
4	19 (15.0)	9 (10.7)	6 (16.7)	4 (57.1)
5	108 (85.0)	75 (89.3)	30 (83.3)	3 (42.9)
mFS on first CT scan (%)				
1–2	15 (11.8)	7 (8.4)	6 (16.6)	2 (28.6)
3–4	112 (88.2)	77 (91.6)	30 (83.4)	5 (71.4)
Treatment modalities				
Microsurgical	60 (47.2)	38 (45.2)	17 (47.2)	5 (71.4)
Endovascular	67 (52.8)	46 (54.8)	19 (52.8)	2 (28.6)
Aneurysm location (%)				
Anterior circulation	103 (81.1)	68 (81.0)	29 (80.6)	6 (85.7)
Posterior circulation	24 (18.9)	16 (19.0)	7 (19.4)	1 (14.3)
Hydrocephalus	66 (52.0)	48 (57.1)	14 (38.9)	4 (57.1)
Intraventricular hematoma	108 (85.0)	75 (89.3)	29 (80.6)	4 (57.1)

Abbreviations: mFS, modified fisher scale; Pts, patients; WFNS, world federation of neurological surgeons.

**FIGURE 3:**
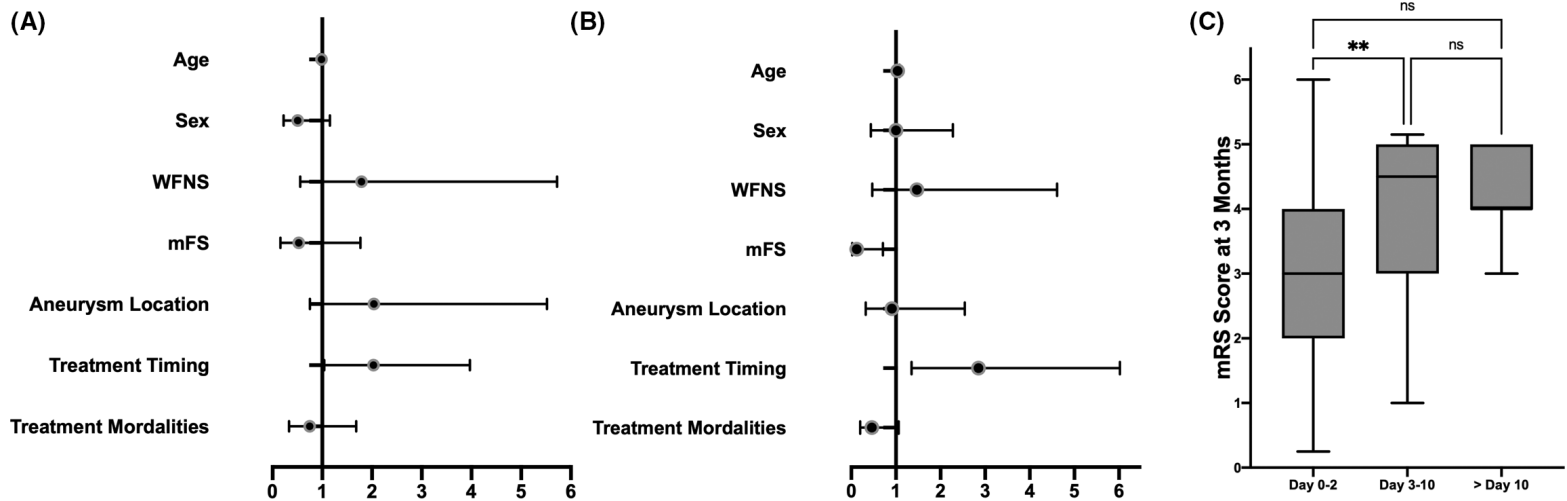
Forest plots for odd ratios of factors associated with (A) DCI and (B) poor prognosis in poor‐grade aneurysmal subarachnoid hemorrhage patients. (C) The prognosis (mRS score) at 3 months for patients treated on days 3–10 and days 11 or later compared with patients treated on days 0–2; *p* value < 0.01, **.

Data on DCI were available for all patients. In the present cohort, the incidence of DCI was 39.4% (50/127) for the whole group, 41.7% (25/60), and 37.3% (25/67) for neurosurgical and endovascular treatment groups, respectively. The risks of DCI for all patients, coiled patients as well as clipped patients, were presented in Table 2. Apart from the treatment timing, no other factors, such as age, sex, clinical condition at admission, amount of blood on the initial CT scan, location of aneurysms, and treatment modalities, were significantly associated with postoperative DCI (Figure 3A). Patients undergoing intermediate surgery (days 3–10) tended to have a higher risk of DCI than those with early surgery (days 0–2) (OR 2.44, 95% CI 1.06–5.64, *p* = 0.036). DCI incidence was higher for clipping group during this period than for coiling group, which approached to a significant difference (*p* = 0.072).

**TABLE 2:** ORs for DCI for patients treated on days 3–10 and days 11 or later compared with patients treated on days 0–2, for all patients and for coiled and clipped patients separately

	*n*/*N* (%)	Crude	Adjusted	Adjusted
		OR (95% CI)	OR (95% CI)	*p* value
All patients	50/127 (39.4)			
0–2 days	27/84 (32.1)	Ref	Ref	Ref
3–10 days	19/36 (52.8)	**2.36 (1.06–5.24)**	**2.44 (1.06–5.64)** ^a^	**0.036** ^a^
>10 days	4/7 (57.1)	2.82 (0.59–13.47)	2.74 (0.49–15.49)^a^	0.253^a^
Clipped patients	25/60 (41.7)			
0–2 days	13/38 (34.2)	Ref	Ref	Ref
3–10 days	10/17 (58.8)	2.75 (0.85–8.90)	3.08 (0.91–10.46)^b^	0.072^b^
>10 days	2/5 (40.0)	1.28 (0.19–8.66)	1.45 (0.178–11.98)^b^	0.732^b^
Coiled patients	25/67 (37.3)			
0–2 days	14/46 (30.4)	Ref	Ref	Ref
3–10 days	9/19 (47.4)	2.06 (0.69–6.17)	1.71 (0.51–5.74)^b^	0.387^b^
>10 days	2/2 (100)	—	—	0.999^b^

*Note*: Boldface type indicates statistical significance.

^a^ Adjusted for age, sex, clinical condition at admission, amount of blood on the initial computed tomography scan, location of aneurysms, and treatment modalities.

^b^ Adjusted for age, sex, clinical condition at admission, amount of blood on the initial computed tomography scan, and location of aneurysms.

Data on the poor prognosis at 3 months was given in Table 3. In total, 71 (71/127, 55.9%) patients have a poor prognosis at 3 months. With increasing time‐lapse of treatment after aSAH, the risk of poor prognosis increased. Old age and a large amount of blood identified during the initial tomography scan were also associated with poor prognosis; however, sex, clinical condition at admission, location of aneurysms, and treatment modalities were not associated with any increased risk of poor prognosis in the analysis (Figure 3B). Patients with intermediate surgery (days 3–10) had the worst prognosis (OR 3.06, 95% CI 1.27–7.39, *p* = 0.013) (Figure 3C). No connection between treatment timing and modalities was identified in neither group. In the subgroup analysis, we divide patients into two categories by the age of 55 years according to our previous study.^19^ For patients aged >55 years and diagnosed with an intraventricular hematoma, the intermediate surgery time (days 3–10) significantly led to poor prognosis (OR 4.30, 95% CI 1.19–15.55, *p* = 0.026 and OR 2.68, 95% CI 1.01–7.10, *p* = 0.048, respectively). Regarding subgroups categorized by preoperative hydrocephalus and perioperative external ventricular or lumbar drainage treatments, no significant difference in the prognosis at 3‐month follow‐up was observed for any treatment timing strategies.

**TABLE 3:** ORs for poor prognosis at 3 months for patients treated on days 3–10 and days 11 or later compared with patients treated on days 0–2 in different subgroups

	*n*/*N* (%)	Crude	Adjusted	Adjusted
		OR (95% CI)	OR (95% CI)	*p* value
All patients	71/127 (55.9)			
0–2 days	39/84 (46.4)	Ref	Ref	
3–10 days	26/36 (72.2)	**3.00 (1.29–6.99)**	**3.06 (1.27–7.39)** ^a^	**0.013** ^a^
>10 days	6/7 (85.7)	6.92 (0.80–60.03)	6.59 (0.66–66.05)^a^	0.109^a^
Clipped patients	38/60 (63.3)			
0–2 days	21/38 (55.3)	Ref	Ref	
3–10 days	13/17 (76.5)	2.63 (0.72–9.56)	3.27 (0.82–13.08)^b^	0.093^b^
>10 days	4/5 (80.0)	3.24 (0.33–31.74)	6.35 (0.49–82.82)^b^	0.158^b^
Coiled patients	33/67 (49.3)			
0–2 days	18/46 (39.1)	Ref	Ref	
3–10 days	13/19 (68.4)	**3.37 (1.08–10.48)**	2.74 (0.75–9.96)^b^	0.127^b^
>10 days	2/2 (100)	——	——	0.999^b^
Age > 55 years	45/75 (60.0)			
0–2 days	27/52 (51.9)	Ref	Ref	
3–10 days	16/20 (80.0)	**3.70 (1.09–12.59)**	**4.30 (1.19–15.55)** ^c^	**0.026** ^c^
>10 days	2/3 (66.7)	1.85 (0.16–21.70)	1.37 (0.09–21.13)^c^	0.823^c^
Hydrocephalus	32/66 (48.5)			
0–2 days	20/48 (41.7)	Ref	Ref	
3–10 days	9/14 (64.3)	2.52 (0.73–8.66)	2.54 (0.62–10.42)^a^	0.194^a^
>10 days	3/4 (75.0)	4.20 (0.41–43.37)	1.39 (0.09–20.75)^a^	0.812^a^
Intraventricular hematoma	59/108 (54.6)			
0–2 days	35/75 (46.7)	Ref	Ref	
3–10 days	20/29 (9.0)	**2.54 (1.02–6.30)**	**2.68 (1.01–7.10)** ^a^	0.048^a^
>10 days	4/4 (100)	—	—	0.999^a^

*Note*: Boldface type indicates statistical significance.

^a^ Adjusted for age, sex, clinical condition at admission, amount of blood on the initial computed tomography scan, and location of aneurysms.

^b^ Adjusted for age, sex, clinical condition at admission, amount of blood on the initial computed tomography scan, and location of aneurysms.

^c^ Adjusted for sex, clinical condition at admission, amount of blood on the initial computed tomography scan, location of aneurysms, and treatment modalities.

## 4 DISCUSSION

Despite a 17% decrease in case fatality in the last three decades for hospitalized aSAH patients, 30‐day mortality unfortunately is still around 35%.^20^ For the poor‐grade aSAH patients, due to the degree of consciousness disorder and severe brain damage, the mortality rate is higher.^12^ Once the aneurysm ruptures and hemorrhages, the recurrence is prone to appear easily (the incidence of rebleeding within 24 h is 4%–13.6%). The mortality rate of rebleeding could reach 70%. And the earlier the rebleeding occurs, the worse the prognosis is. It is known that aneurysms usually deserve to be secured early to prevent rupture and rebleeding consequences. In addition, it is a common clinical sense that patients with vasospasm are more prone to suffer complications from endovascular or open surgical treatment. Both aspects, re‐rupture and unsafe later time windows with vasospasm/DCI, commonly lead to the fact that aneurysms would be treated as early as possible, usually within 24–48 h, unless there is a reason to deviate from that practice. Such reason could be patients with severe early vasospasm are deemed unsafe to undergo surgery, and patients with severe cardiopulmonary decompensation are considered not safe for transfer/surgery. Those reasons are uncommon among good‐grade patients; therefore, most patients can be treated early. However, for poor‐grade aSAH patients, those reasons are commonly presented; thus, the treatment timing remains controversial. Based on the results from the present study, it is rational to fix the ruptured aneurysms as early as it is eligible for treatment in poor‐grade aSAH patients. Together with cohort studies,^8^, ^9^, ^21^ there is robust evidence not to delay treatment, and an early treatment within 48 h might be justified in the decision‐making process.

However, it should be noted that some patients do not receive coiling or clipping in the first visited hospital and are transferred to another hospital for surgical treatment. Time spent during transfer delayed treatment, which fails patients from receiving surgical treatment within 48 h. Historically, clipping between days 5 and 10 is considered the worst period for neurosurgery.^22^, ^23^ It was also applied to endovascular treated patients; Dorhout et al.^4^ reported that patients coiled between 5 and 10 days after the aSAH present a higher risk for DCI than in other treatment periods. The period is in the maximum cerebral vasospasm, and the clipping or embolization inevitably stimulates the vessel and increases the vasospasm and DCI rate. This study also observed that patients treated between 3 and 10 days after the aSAH presented a higher risk for DCI and poor prognosis. It is worthwhile reviewing if it is beneficial for the patient to postpone their surgical treatment until after day 10. Previous studies reported postponing treatment until day 10 or later in patients after hemorrhage may worsen prognosis.^4^, ^5^ In the present study, we observed that patients treated on day 10 or later after aSAH had a worse prognosis than patients treated at the early period, although it did not reach a significant difference due to the small size of the studied patients. Currently, many practitioners prefer early operation after aneurysmal rupture.^4^, ^21^ The potential risk of rebleeding and vasospasm also calls for the early treatment of aneurysms, even for poor‐grade patients. The outcome of this study provides support for current practice. To compare the effect of timing on outcomes, we have confirmed the groups that varied in treatment timing were similar in other baseline demographics. Regular CT angiography for vasospasm and CT perfusion for hypoperfusion were performed to indicate that patients in the later treatment windows do not perform poorer than in the earlier windows. Although it is likely not easy to capture in poor‐grade aSAH patients due to comatose status.

Functional cerebral autoregulation is often impaired in aSAH patients; increasing microcirculatory dysfunction over time can be a causative factor for DCI development and poor outcome.^24^ Maintaining cerebral perfusion pressure could avoid insufficient cerebral blood flow and secondary brain injury.^25^ In addition, a plethora of studies suggest that platelets play a key role in the pathogenesis of DCI after aSAH since platelets are a major factor in micro thrombosis, vasospasm, microvessel constriction, and inflammation after aSAH and may also be an important initiator of cortical spreading depolarization and neurotoxicity.^26^ Thus, early occlusion of the cerebral aneurysm allows for the safer introduction of hypertensive and anti‐platelet therapies to prevent and treat DCI.^27^

The pathological changes after aSAH are associated with the cerebrospinal fluid (CSF) dynamic disorder involving CSF production, movement, and drainage.^28^ Early microsurgical interventions could wash cisternal blood; lumbar cistern drainage and external ventricular drainage after coiling could also accelerate blood CSF clearance and improve clinical outcomes in SAH patients. Based on the results of various preclinical SAH models, the potential mechanism underlying hydrocephalus might be the CSF hypersecretion from the choroid plexus.^29^ And more than half of aSAH patients with intraventricular hematoma developed hydrocephalus.^30^ The CSF dynamic modulation after aSAH might be especially applied in patients with intraventricular hematoma. The result of subgroup analysis in the present study presented that patients with intraventricular hematomas might benefit from the early intervention of the aneurysm. Endothelium impairment after aneurysm rupture disrupts the blood–brain barrier, further driving water influx to the parenchymal side and contributing to cerebral edema formation. Cerebral edema with increased intracranial pressure over a longer period also contributes to poor outcomes. Thus, poor‐grade aSAH patients very frequently require CSF diversion that can help prevent intracranial pressure peaks that can remain undetected with mere CT follow‐up; it can also help clear the CSF from the blood. Irrespective of the importance or advantage of external ventricular or lumbar drainage treatment has been widely recognized^1^; not all patients received external ventricular or lumbar drainage treatment to drain hemorrhage during open surgery or after coiling in the present study. Our data demonstrated no significant difference between the poor prognosis rate in patients with and without external ventricular drainage or lumbar drainage treatment (30/59, 50.8% vs. 41/68, 60.3%). Thus, to achieve a better outcome in poor‐grade aSAH patients, although treatment to drain hemorrhage indeed play a certain role, the early intervention of the aneurysm may be essential.

Since the first detachable coil occluded an aneurysm in 1991,^31^ endovascular treatment is now an established treatment for aneurysms. The International Subarachnoid Aneurysm Trial demonstrated the probability of death or dependency was significantly lower in the endovascular group than in the neurosurgical group.^32^ However, poor‐grade aSAH patients accounted for only 5% of the entire cohort in this trial. Thus, the above conclusions might not fully apply to patients with poor‐grade aSAH. It is common knowledge that open surgery can wash cisternal blood, eliminating a possible factor in the development of vasospasm. In contrast, although there is no significant difference, our data showed that the incidence of DCI and poor prognosis is higher in the microsurgery group than in the endovascular group. In poor‐grade aSAH patients, further injury to the brain from craniotomy might outweigh the benefit of washing local cisternal blood. Treatment of early brain injury may be the main measure to improve patients' prognosis.^33^ Thus, an external ventricular or lumbar drainage treatment to drain hemorrhage after coiling seems to be a better choice for patients who are not at risk of herniation. Ideally, a new randomized trial would be needed to provide definite answers to direct decisions of surgical intervention in poor‐grade aSAH, thus improving the clinical prognosis for patients.

This large series of patients was evaluated by neurosurgeons trained in aSAH pathology with access to all medical information. Although this was an important strength of the study, the LongTEAM trial is an observational study; patients were not randomized for the timing of treatment, which led to large differences in the sample size of each surgery timing category. Although for the baseline demographics shown, the groups appear similar, the differences in other actual relevant factors that were not assessed or not displayed here between the time pretreatment time window may bias the result. Furthermore, the number of patients is too small to garner a meaningful conclusion about late treatment.

## 5 CONCLUSION

In summary, our results indicate that aneurysm treatment between 3 and 10 days after aSAH is associated with a worse prognosis, regardless of treatment modality. Further subgroup analysis suggested an early intervention for patients' potential benefits, which promoted a policy for early intervention as soon as possible, especially for patients older than 55 years or with an intraventricular hematoma. Based on these results, postponing treatment was not recommended in patients eligible for aneurysm treatment within 48 h after hemorrhage.

## FUNDING INFORMATION

This study is supported by the National Key Research and Development Program of China (2020YFC2004701). National Natural Science Foundation of China (82071302).

## CONFLICTS OF INTEREST

The authors declare no competing interests.

## Data Availability

The data that support the findings of this study are available from the corresponding author upon reasonable request.
